# Extraskeletal myxoid chondrosarcoma: a case report

**DOI:** 10.11604/pamj.2023.44.199.39846

**Published:** 2023-04-25

**Authors:** Anass Haloui, Nassira Karich, Asmae Aissaoui, Nada Akouh, Chaimae Bekhakh, Omar Mokhtari, Najib Abdeljaouad, Amal Bennani

**Affiliations:** 1 Laboratory of Pathological Anatomy, Mohammed VI University Hospital, Faculty of Medicine and Pharmacy of Oujda, Mohammed First University, Oujda, Morocco,; 2 Department of Trauma and Orthopaedics, Mohammed VI University Hospital, Faculty of Medicine and Pharmacy of Oujda, Mohammed First University, Oujda, Morocco

**Keywords:** Extraskeletal myxoid chondrosarcoma, soft tissue, sarcoma, case report

## Abstract

Extraskeletal myxoid chondrosarcoma is a rare mesenchymal neoplasm of uncertain differentiation, characterized morphologically by abundant myxoid stroma, a multinodular growth pattern, and uniform cells arranged in strands, clusters, and reticular networks. It usually occurs in adults in the fifth decade, most often in the deep soft tissues of the proximal extremities. The molecular hallmark of this tumor, present in over 90% of cases, is the fusion of NR4A3 with EWSR1 at 22q12.2 or TAF15 at 17q12. Many other tumors with uniform tumor cells embedded in a myxoid matrix can mimic Extraskeletal myxoid chondrosarcoma, and the distinction can be difficult, often requiring immunohistochemistry and/or molecular testing. We herein report the case of an Extraskeletal myxoid chondrosarcoma that occurred in a 74-year-old woman who consulted for a slowly enlarging thigh mass, while highlighting the key morphologic, immunohistochemical, and molecular features of this rare type of soft tissue sarcoma, as well as a summary table gathering diagnostic features of relevance to the differential diagnosis.

## Introduction

Extraskeletal myxoid chondrosarcoma (EMC) is a rare malignant mesenchymal neoplasm of uncertain differentiation, characterized by abundant myxoid matrix, multilocular architecture, and uniform cells arranged in cords, clusters, and reticular networks [1]. It occurs primarily in soft tissue and rarely, if ever, in bone. In spite of its name, there is no evidence of cartilaginous differentiation, and this designation is attributable to certain ultrastructural features and properties of its matrix, which is Alcian blue positive and partially hyaluronidase resistant, classically cartilaginous features, hence the term chondrosarcoma [2]. We herein present a case of an EMC occurring in 74-year-old woman, while highlighting key morphologic, immunohistochemical, and molecular features relevant to the diagnosis.

## Patient and observation

**Patient information:** a 74-year-old woman with history of diabetes and arterial hypertension, presented with a painless mass on the inner part of the left thigh, evolving for 4 years.

**Clinical findings:** physical examination revealed a painless mass of hard consistency, located in the inner part of the left mid-thigh.

**Timeline of current episode:** on September 2022, an X-ray and MRI of the left thigh were performed. Few days after, a biopsy followed by the resection of the mass were performed. A chest X-ray was also performed.

**Figure 1:**
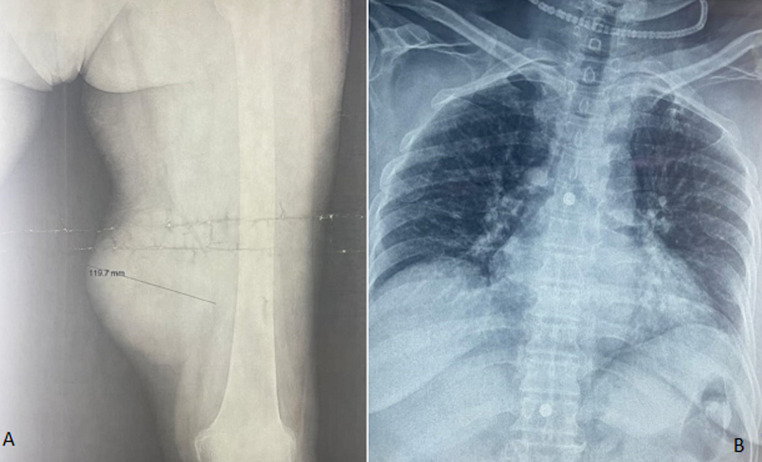
A) X-ray of the left thigh showing an oval opacity in the soft parts located in the inner face of the mid-thigh; B) chest X-ray showing pulmonary nodules of metastatic appearance

**Figure 2:**
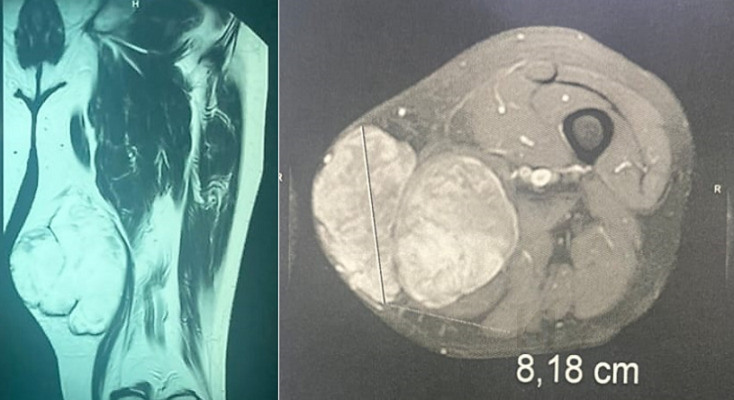
MRI of the thigh showing a well limited soft tissue mass, roughly rounded, with polylobed contours, measuring 8cm in great axis, pushing the medial rectus muscle, respecting the femur and the vascular-nervous bundle

**Figure 3:**
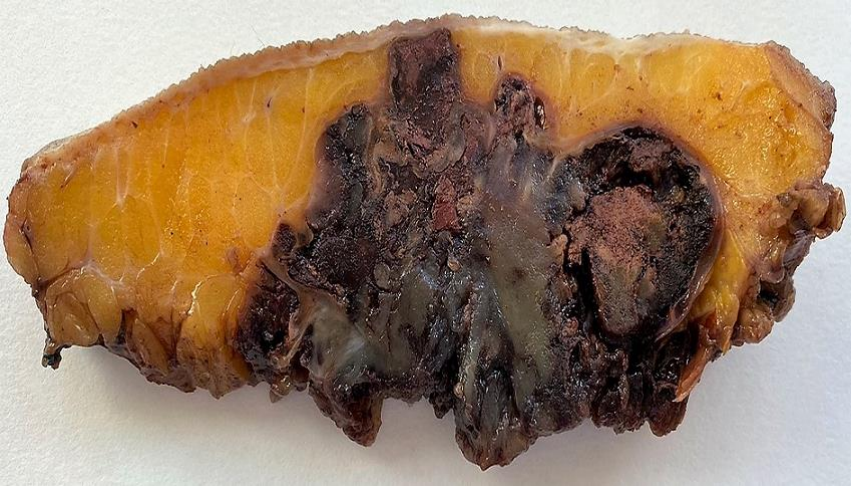
macroscopic appearance of the tumor, showing a soft tissue encapsulated mass with polylobed contours, displaying multiple lobules of gelatinous appearance, separated by fibrous septa with prominent hemorrhage and necrosis

**Figure 4:**
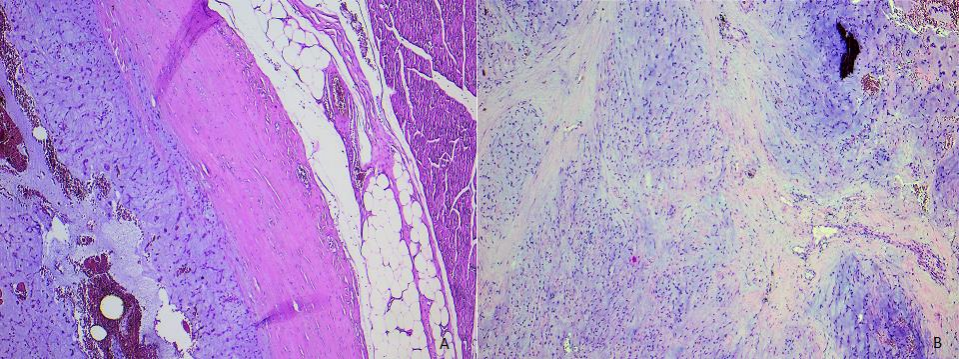
A) low power view showing a soft tissue tumoral proliferation, well limited at periphery by a fibrous capsule; B) multinodular growth pattern with delicate fibrous septas (Hematoxylin and eosin staining, magnification x4)

**Figure 5:**
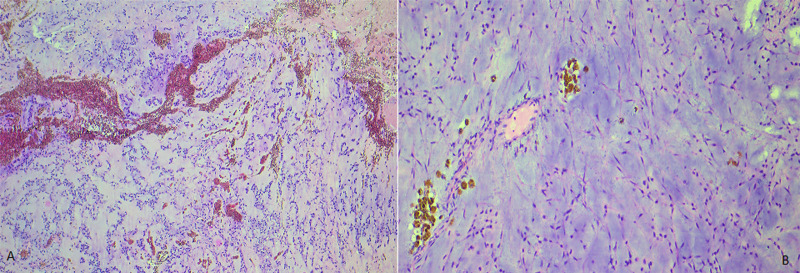
A) low magnification view showing a striking myxoid background with prominent intra-tumoral hemorrhage (Magnification x4); B) strands and cords of interconnected tumor cells embedded within the myxoid matrix, with focal hemosiderin deposition (magnification x10)

**Figure 6:**
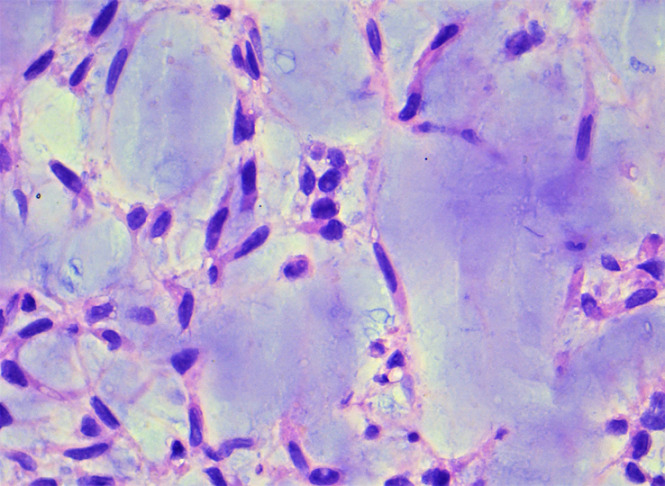
uniform tumor cells provided with bland ovoid nuclei and interconnected by delicate cytoplasmic processes (magnification x40)

**Figure 7:**
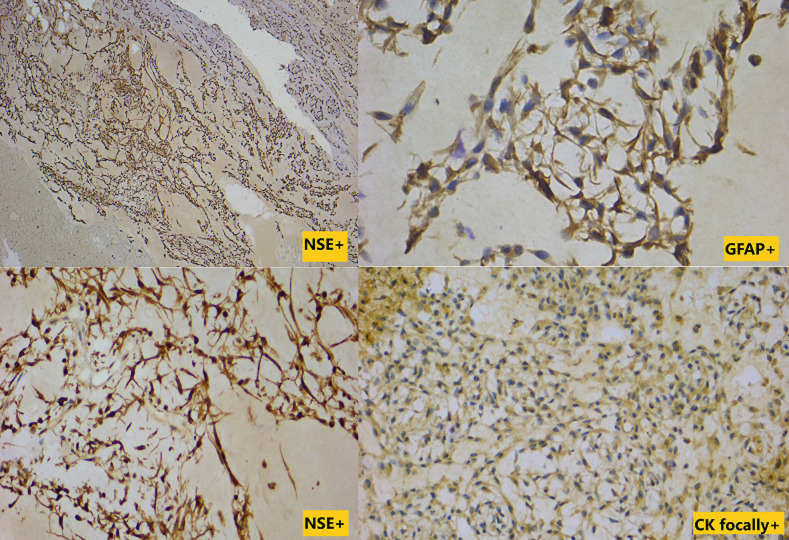
tumors cells showing diffuse expression of GFAP and NSE while EMA and cytokeratin were expressed focally

**Diagnostic assessment:** the X-ray of the thigh showed an oval opacity in the soft parts of the inner face of the left mid-thigh. The Chest X-ray revealed the presence of pulmonary nodules of metastatic appearance (Figure 1). The MRI revealed a well limited soft tissue mass, roughly rounded, with polylobed contours, in T1 hypo signal and T2 heterogeneous hyper signal, measuring 8cm in great axis, pushing the medial rectus muscle, respecting the femur and the vascular-nervous bundle (Figure 2). A biopsy of the mass followed by its resection were performed. The gross examination of the resection specimen revealed an encapsulated mass with polylobed contour, measuring 8x7cm, displaying at cut multiple lobules of translucent gelatinous appearance, separated by fibrous septa, with prominent hemorrhage and necrosis (Figure 3). The histological findings in the biopsy and the resection specimen were identical, revealing a tumoral proliferation well limited by a fibrous capsule at periphery, showing a multinodular growth pattern, made of strands and cords of bland looking cells, embedded within copious myxoid matrix (Figure 4). The stroma is densely myxoid and hypo vascular, with prominent hemorrhage and focal hemosiderin deposition (Figure 5). The tumor cells are provided with small ovoid nuclei, and acidophilic cytoplasm, occasionally vacuolated, emitting cytoplasmic projections interconnecting the cells (Figure 6). Immunohistochemically, the tumors cells diffusely expressed Glial fibrillary acidic protein (GFAP) and neuron-specific enolase (NSE). While Epithelial membrane antigen (EMA) and Cytokeratin were expressed focally (Figure 7).

**Diagnosis:** the results were consistent with an Extraskeletal myxoid chondrosarcoma, based on morphological and immunohistochemical features, along with clinical and radiological correlation.

**Therapeutic interventions:** the patient underwent chemotherapy followed by a resection of the mass.

**Follow-up and outcome of interventions:** to date, the patient remains asymptomatic and in excellent clinical condition, with no reported complications.

**Patient perspective:** “I feel well”.

**Informed consent:** the patient gave informed consent.

## Discussion

EMC is uncommon, accounting for < 1% of all soft tissue sarcomas. It mainly occurs in adults between the fourth and sixth decades (median age of 50 years old) with a slight male predilection. Children and adolescents are exceptionally affected. Clinically, it presents as a slowly growing mass in the deep soft tissues, with a duration ranging from few weeks to several years [3], often associated with pain and tenderness. It usually arises in the proximal extremities and limb girdles, with the thigh being the most frequent site. Other rare localization has been described in the literature such as the trunk, retroperitoneum, mediastinum, central nervous system, and vulva [4]. It should be noted that morphologically similar tumors can occur in bone. Therefore, radiography, CT scan or MRI are required to determine the soft tissue origin, even though they provide no distinguishing features from other types of soft tissue sarcomas [3]. In our case, although the localization in the thigh was consistent with literature, the age of the patient (74-year-old) was higher than the median age reported in literature.

Macroscopically, most EMCs are well circumscribed, surrounded by a fibrous pseudocapsule. The tumor size is variable ranging from few centimeters to 30 cm, with a median size of 4 to 7cm. On section, the tumor is soft to firm, of grey to tan-brown color, displaying a lobulated appearance comprising gelatinous areas separated by fibrous septa, with possible cystic cavities, hemorrhage, and necrosis. The macroscopic examination in our case was consistent with the macroscopic appearance reported in the literature, with a soft tissue mass measuring 8cm in great axis, encapsulated by a fibrous capsule, dissected by fine fibrous septa delimiting several gelatinous lobules, with hemorrhage foci. Microscopically, a multinodular architecture with a striking myxoid background is usually noticeable at low magnification. The tumor is made of hypocellular nodules with increased cellularity at the periphery, separated by delicate fibrous septa, containing cords, clusters, and delicate strands of uniform interconnected cells. The tumor cells are provided with small, round to oval nuclei, evenly distributed chromatin, inconspicuous nucleoli, and modest amount of deeply eosinophilic to vacuolated cytoplasm, set in an abundant pale blue myxoid matrix composed of sulfated mucopolysaccharides [4]. The stroma is hypo-vascular with frequent areas of prominent hemorrhage and hemosiderin deposition. Frank cartilaginous differentiation is extremely rare, if never present. A cellular variant of EMC characterized by increased cellularity has been described. Some cases may display rhabdoid features characterized by cells with large paranuclear hyaline inclusions, indicating aggressive behavior [5]. Other cases may display small round cells mimicking an extraskeletal Ewing family of tumors. It should be noted that typical EMC may be associated with or progress to a high-grade pleomorphic sarcoma, referred to as dedifferentiated EMC [3]. The histological findings in our case were consistent with literature. The myxoid background was striking at low magnification, comprising strand, chords, and clusters of uniform cells, provided with ovoid nuclei with fine chromatin, and interconnected by delicate cytoplasmic processes. Prominent hemorrhage and focal hemosiderin deposition were also present.

Immunohistochemically, EMC consistently display strong expression of vimentin. Twenty percent (20%) of cases are positive for PS100 [6], which is far from what one might expect from a true cartilaginous neoplasm. Occasional cases are EMA positive. Numerous authors have described focal expression of neural/neuroendocrine markers such as NSE, Synaptophysin, Chromogranin and CD56. Like many other types of sarcomas, rare cases display immunoreactivity for Cytokeratin. Few cases may express CD117 [7]. The tumor cells in our case diffusely expressed GFAP and NSE, and focally expressed EMA and Cytokeratin. Cytogenetically, EMC are one of the sarcomas with a consistent karyotypic abnormality. In most instances, the genetic hallmark of this tumor is a translocation t(9;22) (q22;q12) leading to the gene fusion EWSR1-NR4A3. Less commonly, a variant translocation t(9;17) is present, which results in RBP56-NR4A3 gene fusion [8]. Molecular tests using paraffin-embedded tissues, such as FISH or RT-PCR, can detect these alterations and thus be of great help in confirming the diagnosis in challenging cases. In our case, molecular testing was not carried out given the characteristic morphological appearance.

Drawing a clear line of demarcation between EMC and other myxoid or chondroid-like lesions may be challenging on the basis of morphologic appearance alone, and immunohistochemistry or even molecular testing is often required, along with both clinical and radiological correlation. Table 1 lists the major tumors morphologically overlapping with EMC. The main differential diagnosis is benign and malignant myoepithelioma of soft tissue, which can display a multinodular growth pattern, with cords of cells embedded in a dense myxoid background, closely resembling EMC. However, the absence of ductal architecture, along with the presence of dense sclerosis, hemorrhage and hemosiderin pigment surrounding nodules of tumor are highly suggestive of EMC. Furthermore, myoepithelioma is consistently positive for Keratin, EMA and PS100, unlike EMC. Recently, it has been showed that 50% of myoepitheliomas harbor EWSR1 aberrations, adding further confusion to the distinction from EMC. Myxoid variant of chondroma can be deceptive, especially when displaying plump immature-appearing cells in a myxoid background. As a rule, they occur in the soft tissues of hands and feets, unusual locations for EMC, and can be recognized by the presence of more mature cartilaginous areas at the peripheral portion of the tumor. Chondromyxoid fibroma occurs mainly in metaphysis of long bones, rarely extending into soft tissue as secondary implantation. This tumor also displays a lobular architecture. However, it can be readily distinguished from EMC by the greater degree of cellular pleomorphism, as well as the disparity of cellular distribution within tumor lobules, with hypocellular centers comprising stellate cells and hypercellular periphery consisting of fibroblast-like cells and multinucleated osteoclast-like giant cells. In Ossifying fibro myxoid tumor, the lobulated architecture and arrangement of the neoplastic cells into cord-like structures bear some resemblance to extraskeletal myxoid chondrosarcoma, but the stroma varies between myxoid and collagenous, and the neoplastic cells have less eosinophilic cytoplasm than those of myxoid chondrosarcoma. Chordoma, especially in its myxoid form, may enter the differential diagnosis, but this diagnosis is unlikely if the tumor occurs outside of its usual location in the sacrococcygeal region, the base of the skull, or the cervical spine. Furthermore, EMC shows no radiographic evidence of bone involvement and lacks physaliphorous tumor cells. In our case, the most problematic tumor that had to be ruled out was myoepithelioma, since pulmonary metastatic nodules can also be found in this condition. However, the absence of major ductal arrangement of tumor cells, as well as the presence of foci of hemorrhage and hemosiderin deposits, pleaded in favor of an EMC. Late recurrence and metastasis are common in EMC. The most common metastatic sites are lung, soft tissue, and lymph nodes. a large retrospective serie [9] have reported a 5-year survival rate of 82-90%, with older age, large tumor size (>10cm), and proximal location being adverse prognostic factors. Radical excision of the mass, with or without adjuvant radiotherapy, seems to be the treatment of choice, while chemotherapy, reserved for metastatic cases, has not been shown to be effective [7].

**Table 1:** main differential diagnosis of extra skeletal myxoid chondrosarcoma

Diagnosis	Age	Frequent sites	Macroscopy	Histology	Immunohistochemistry
Myxoid variant of extra skeletal chondroma	Adults 30 -60 years old	Hands and feets	well-demarcated, oval-round masse. < 3 cm	Mature hyaline cartilage arranged in distinct lobules with sharp borders. Myxoid changes possible.	PS100 + consistently
Chondromyxoid fibroma	young adults 10-25 years old	Metaphysis of Long bones. Rarely in soft tissue as secondary implantations	Lobulated mass. Cut surface is firm, yellowish white or tan, with variable myxoid areas.	Lobulated architecture. hypocellular centers (Stellate cells) and hypercellular periphery (fibroblast-like cells and scattered multinucleated giant cells.) Myxo-chondroid matrix	PS100 + in stellate cells SMA focally+ in spindle cells
Chordoma	Adults 50-60 years old	bones of axial skeleton. May rarely extend into soft tissue.	Lobular solid mass. Gelatinous at cut.	Lobulated architecture with fibrous septa. Physaliphorous cells arranged in cord and nests, embedded within myxoid matrix	Coexpressession of PS100, brachyury , EMA and Cytokeratin
Ossifying fibro-myxoid tumor	Median age 50 years	Subcutis or within skeletal muscle of the extremities	Median size of 4cm. Well limited. Thick fibrous pseudocapsule The cut surface is often glistening, white to tan	Frequent capsular ossification. Lobulated growth pattern. Cords, nests, and trabeculae of ovoid cells with bland nuclei and scant pale eosinophilic cytoplasm. The stroma varies between myxoid and collagenous.	PS100+, CD10+ Desmine +(50%) EMA+/-, CK+/-
Myoepithelioma	young adults 20-40 years.	Deep soft tissue of the extremities, usually muscles of the thigh, calf, upper, arm Head and neck possible.	Nodular mass. Median size 3 to 7cm Cut surface is glistening, myxoid, or gelatinous. Yellow white to tan	well circumscribed with fibrous pseudo capsule Cords/ trabeculae of epithelioid or spindled cells. Ductal differentiation + Stroma is variable: myxoid, chondromyxoid or extensively hyalinized. Marked nuclear atypia in myoepithelial carcinoma	CK+, EMA+, PS100+ SMA and P63 + in 50%
Myxoid liposarcoma	30-50 years	Deep soft tissue of extremities, thigh+	Well-circumscribed. Multilobular. yellow-fatty to gelatinous.	Prominent myxoid stroma. “Chicken-wire” vasculature. signet-ring lipoblasts.	PS100 + in most cases MDM2-
Intramuscular myxoma	40-70 years	Thigh, shoulder, upper arm	Poorly circumscribed merging with the surrounding skeletal muscle. 5 to 10 cm. Glistening,gelatinous, gray-white at cut.	Hypocellular Bland spindled or stellate cells separated by abundant myxoid stroma. Paucity of vascular structures	CD34+, Vimentine+ PS100-

## Conclusion

The morphology of EMC is quite characteristic, and the diagnosis is generally easy to make if one is familiar with the entity. However, other soft tissue tumors can be morphologically confusing, and the use of immunohistochemical and molecular studies, along with a correlation with clinical and radiological findings may be necessary.
